# Prognostic Value of SGK1 and Bcl-2 in Invasive Breast Cancer

**DOI:** 10.3390/cancers15123151

**Published:** 2023-06-11

**Authors:** Umaima Al-Alem, Garth H. Rauscher, Qais Al Alem, Andre Kajdacsy-Balla, Abeer M. Mahmoud

**Affiliations:** 1 Division of Epidemiology and Biostatistics, School of Public Health, The University of Illinois at Chicago, Chicago, IL 60612, USA; ualale2@uic.edu (U.A.-A.); garthr@uic.edu (G.H.R.); qais.al.alem@gmail.com (Q.A.A.); 2 Department of Pathology, College of Medicine, The University of Illinois at Chicago, Chicago, IL 60612, USA; aballa@uic.edu; 3 Division of Endocrinology, Diabetes, and Metabolism, Department of Medicine, College of Medicine, The University of Illinois at Chicago, Chicago, IL 60612, USA; 4 Department of Kinesiology and Nutrition, College of Applied Health Sciences, The University of Illinois at Chicago, Chicago, IL 60612, USA

**Keywords:** breast cancer, tissue microarrays, GCR, SGK1, Bcl-2, prognosis, survival, hormone receptor positive, breast cancer survival

## Abstract

**Simple Summary:**

We have previously shown that the glucocorticoid receptor (GCR) protein was reduced in invasive breast carcinoma compared to normal breast tissue. Here, we evaluated the level of serum/glucocorticoid-regulated kinase 1 (SGK1) and B-cell lymphoma 2 (Bcl-2) levels in the corresponding primary breast cancer tissue. SGK1 was higher and Bcl-2 was lower in breast cancer tissue compared to normal breast tissue. Similar to previous reports, we found that the expression of the Bcl-2 protein was associated with longer survival. We observed a correlation between the expression of the GCR and the Bcl-2 protein. The expression of the Bcl-2 protein was higher among cases who self-reported their race and ethnicity as non-Hispanic Black people.

**Abstract:**

It is crucial to understand molecular alterations in breast cancer and how they relate to clinicopathologic factors. We have previously shown that the glucocorticoid receptor (GCR) protein expression was reduced in invasive breast carcinoma compared to normal breast tissue. Glucocorticoids, signaling through the GCR, regulate several cellular processes via downstream targets such as serum/glucocorticoid-regulated kinase 1 (SGK1) and B-cell lymphoma 2 (Bcl-2). We measured the expression of SGK1 and Bcl-2, in respective breast cancer tissue arrays, from a multiracial cohort of breast cancer patients. Higher cytoplasmic SGK1 staining was stronger in breast cancer tissue compared to normal tissue, especially in hormone receptor-negative cases. Conversely, the expression of cytoplasmic Bcl-2 was reduced in breast cancer compared to normal tissue, especially in hormone receptor-negative cases. Bcl-2 staining was associated with the self-reported racial/ethnic category, an earlier clinical stage, a lower histological grade, and a higher survival rate. Bcl-2 expression was associated with longer survival in models adjusted for age and race (HR = 0.32, 95% CI: 0.15, 0.65), and Bcl-2 expression remained strongly positively associated with protection from breast cancer death, with additional adjustments for ER/PR status (HR = 0.41, 95% CI: 0.2, 0.85). SGK1 and Bcl-2 may play biological roles in breast cancer development and/or progression.

## 1. Introduction

Breast cancer is a multifactorial disease whose onset and progression are influenced by genetic, epigenetic, and environmental factors, which affect normal cell proliferation, apoptosis, and survival, leading to tissue invasion and metastases. Despite a decrease in overall incidence and mortality between racial and ethnic groups in the United States, Black and Hispanic women are still more likely than their White counterparts to present at an earlier age, with different characteristics of breast cancer, such as later stages of breast cancer and aggressive tumors that have poor prognoses [1].

Glucocorticoid signaling pathways are situated at focal points regulating cellular processes such as apoptosis, inflammation, mammary development, and tumorigenesis [2,3]. The functional isoform glucocorticoid receptor alpha (GCR) exists mainly in the cytoplasm and mediates glucocorticoid signaling. Almost all human tissues express GCR in a cell-specific fashion [3,4]. When bound to glucocorticoids, GCR moves into the nucleus, where it controls the transcription of numerous genes. The expression level, subcellular localization, and interactions with other genes all play a role in regulating GCR activity. The GCR nuclear receptor connects environmental factors to physiological, metabolic, and pathological processes by acting as an endocrine and environmental status sensor.

The altered response to GCR has been associated with the pathogenesis of several diseases, such as metabolic syndrome [5], cardiovascular disease [6], rheumatoid arthritis [7], depression [8], and sporadic breast cancer among Caucasian women [9]. Evading apoptosis and altering energy metabolism are among the hallmarks of cancer, and glucocorticoids, signaling through GCR and downstream target genes, have been shown to regulate both processes. Glucocorticoids are used to induce anti-inflammation through apoptosis [10]. However, GCR has been shown to induce pro- and anti-apoptotic effects in a tissue-specific manner [11]. Glucocorticoids regulate adipose tissue’s differentiation, function, and distribution, especially in visceral obesity [12]. Defects in GCR-mediated signaling could be a link between apoptosis, metabolic syndrome, and aggressive breast cancer.

Our previous study showed reduced GCR protein expression in breast cancer tissue [13]. In this study, our main objective is to understand if there is a correlation between GCR reduction and its two downstream target genes, serum and glucocorticoid-regulated kinase-1 (SGK1) [14] and B-cell lymphoma 2 (Bcl-2), from the respective tissue arrays [15]. SGK1 is a serine/threonine kinase that belongs to the protein kinase AGC family and participates in many cellular processes. SGK1 dysregulated expression was observed in hypertension, cancer, autoimmunity, and neurodegenerative disorders [16,17], and it was reported to suppress apoptosis and cellular adhesiveness in breast cancer cell lines [18,19]. Bcl-2 is a member of the anti-apoptotic Bcl-2 family of proteins. Its expression has been linked to favorable prognosis, hormone receptor positivity, lower histological grades, and better survival in breast cancer patients [20].

GCR and its downstream targets, SGK1 and Bcl-2, are critical for several biological processes influencing breast cancer growth and progression. Building on our previous finding of reduced GCR in invasive breast cancer compared to normal tissues, we sought to investigate the expression of SGK1 and GCL2 in the corresponding tissue microarrays and correlate these histological findings with clinicopathological parameters, including age, race, body mass index (BMI), menopausal status, histological subtype and grade, clinical stage, molecular subtype, and hormonal status, as well as survival rates. The main hypothesis is that the downregulation of GCR could alter SGK1 and Bcl-2 levels, which may contribute to the progression or aggression of breast cancer. We used tissue microarray technology to investigate breast cancer cases with defined clinical characteristics. The originality of this study lies in evaluating the prognostic value of GCR, SGK1, and Bcl-2 in the Breast Cancer Care in Chicago (BCCC) cohort, a multiethnic population of incident breast cancer cases with archived biological samples and linked clinical, genetic ancestry, survival, and sociodemographic data.

## 2. Materials and Methods

### 2.1. Study Population and Tissue Microarray Construction

**Figure 1:**
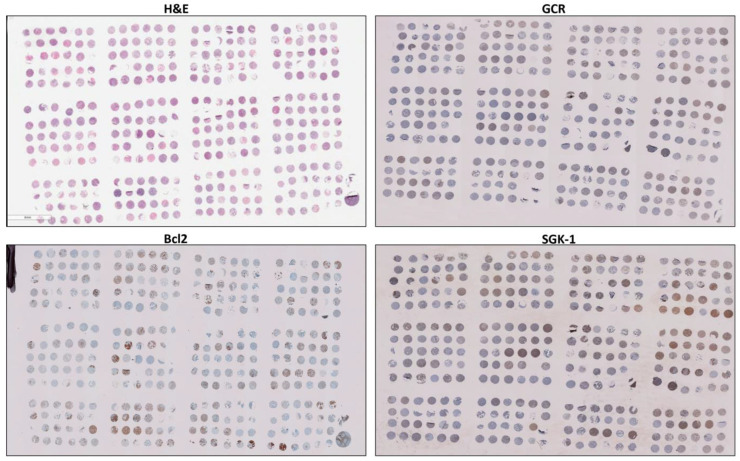
Representative images of one of the tissue microarrays (TMAs) stained for hematoxylin and eosin (H&E) and the targeted proteins in the study: GCR, SGK1, and Bcl-2.

There were three tissue microarrays constructed from paraffin-embedded surgical samples of tumors before the initiation of radiation, chemotherapy, or hormone therapy from the ‘Breast Cancer Care in Chicago (BCCC)’ study, a population-based cross-sectional study of breast cancer cases with primary invasive breast cancer, diagnosed between 2005 and 2008 in Chicago, conducted by the UIC Center for Population Health and Health Disparities. Pathologists at the University of Illinois Hospital initially diagnosed cases as invasive breast cancer, fibroadenoma (benign breast tumors of both glandular and stromal tissue), or non-tumoral breast tissues during the routine analysis of surgically obtained specimens (the whole sections before TMA construction). Then, three pathologists evaluated the cores independently and confirmed the diagnosis, as described in our previous publications [13,21]. The methods used to design the issue microarray have been described, in detail, in our previous reports [13,21]. Briefly, the tissue microarrays (TMA) contained tumor tissue from 280 cases, 26 normal breast tissues from unaffected women obtained by reduction mastectomy procedures, and 6 fibroadenomas from UIC Medical Center in the tissue microarray. On the TMA, every case was represented by three cores from three distinct tumor sections for that subject. Cores from corresponding patients were randomly distributed across TMAs to avoid batch or position effects. Scores reflected the calculated mean of the three cores for each case. Figure 1 depicts the hematoxylin and eosin (H&E) staining and labeling of the targeted proteins in one of the tissue microarrays.

### 2.2. Immunohistochemical Staining

TMAs were serially sectioned, deparaffinized, and rehydrated. This was followed by using the suitable antigen retrieval technique. As previously described, histological sections were then incubated with the proper primary antibodies (listed in Table 1), followed by the proper secondary antibodies. Finally, sections were stained with 3,3-diaminobenzidine (DAB) and the counterstain, hematoxylin [13]. Respective TMAs were stained for each protein separately.

### 2.3. Immunohistochemical Scoring and Molecular Breast Tissue Subtyping

A trained pathologist performed the scoring without knowledge of the case outcomes. The expression of SGK1 and GCR was evaluated based on the percentage of positive tumor cells and the intensity of the stain. The H score is calculated from the percentage of cells (0 to 100%) in each intensity category (0, 1+, 2+, and 3+). The final H score is a continuous scale between 0 and 300. A mean H score of the triplicate cores was used. For the scoring of Bcl-2, a semiquantitative scale was used, which classifies tumors from 0 to 3, according to the number of stained tumor cells and the intensity of the reaction, where 0 = total negative, 1 = <20% of cells show reliable staining, 2 = 20–80% show strong staining, and 3 = all cells are strongly positive. Molecular subtypes, determined according to ER, PR, HER2, CK 5/6, and EGFR expression, were performed as previously described [13]. Breast cancers were classified as Luminal A (ER+ or PR+/HER2-), Luminal B (ER+ or PR+/HER2+), HER2 enriched (ER-/PR-/HER2+), and triple-negative (ER-/PR-/HER2-).

### 2.4. Statistical Analysis

The primary response variables were immunohistochemical scores for GCR, SGK1, and Bcl-2. Immunohistochemical scores were dichotomized based on the median H score (GCR = 17, SGK1 = 30, and Bcl-2 = 0) and used to assess the correlation with our outcome variables: stage, grade, histological subtype, and hormone receptor status (each abstracted from patient medical records). The stage of diagnosis was classified using the categories of the American Joint Committee on Cancer (AJCC) of 0, 1, 2, 3, and 4. The later stage was defined as stages 2, 3, and 4 vs. 0 and 1. The histological grade was determined to be low, intermediate, and high. A higher grade was defined as a high grade versus a low/intermediate grade. The ER/PR status was positive if the tumor contained estrogen (ER) and/or progesterone (PR) receptors and negative without both receptors. The Molecular Subtypes were classified as Luminal A, Luminal B, HER2 positive, and triple negative. Race/ethnicity was defined by separate self-identification, and it was classified as non-Hispanic White, non-Hispanic Black, and Hispanic. To compare clinical and histopathological characteristics, we performed the χ2 test for dichotomous variables and a one-way ANOVA model for continuous variables. We also fit logistic regression models to estimate odds ratios and 95% CI. The period from the date of diagnosis to death from any cause, or the date of the final follow-up, was used to determine overall survival (OS). The term “breast cancer-specific survival” (BCSS) refers to the period between the date of diagnosis and the breast cancer-related mortality or the date of the final follow-up. The Kaplan–Meier approach was used to estimate survival curves, and a log-rank test was used to assess the significance of the variation in survival curves. The hazard ratio (HR) and 95% confidence interval were calculated using the Cox proportional hazards model (CI). Every *p*-value that is presented is two-sided, and a *p*-value of 0.05 or below was regarded as statistically significant. Stata version 17 was used to conduct statistical analyses (College Station, TX, USA).

## 3. Results

### 3.1. Baseline Characteristics of the BCCC Subcohort in the Tissue Microarray Study

**Figure 2:**
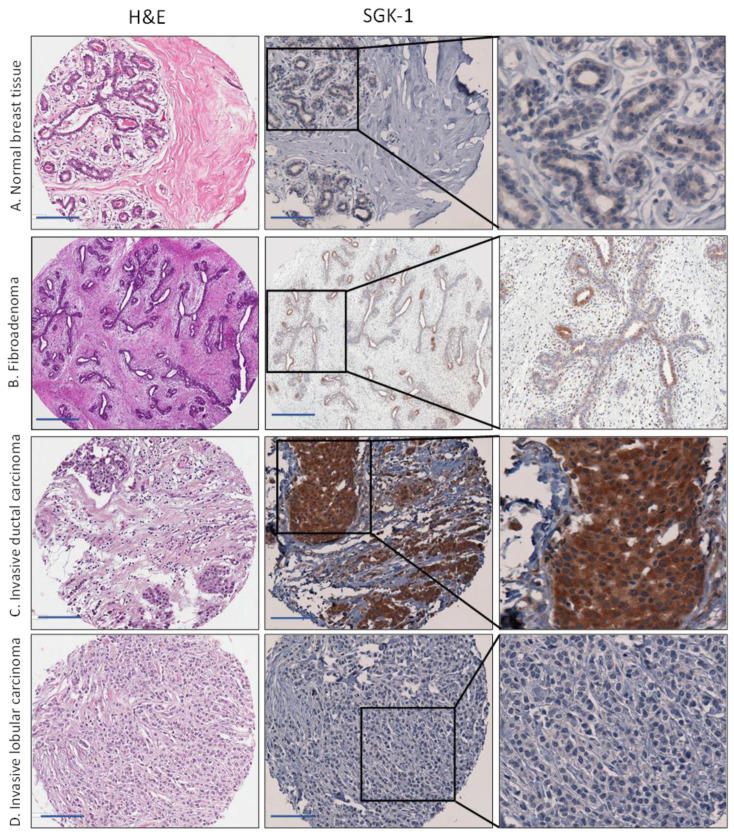
Immunohistochemical staining for serum/glucocorticoid regulated kinase 1 (SGK1) in representative cases of (**A**) normal breast tissue, (**B**) benign breast lesion (fibroadenoma), (**C**) invasive ductal carcinoma, and (**D**) invasive Lobular carcinoma. Scale bar is 50 µm.

**Figure 3:**
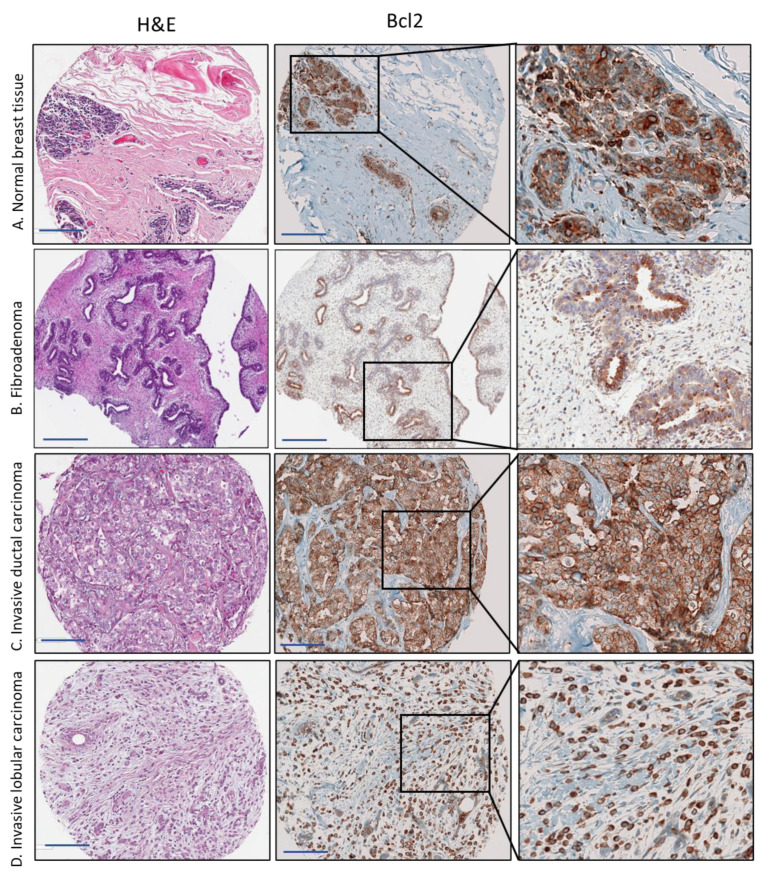
Immunohistochemical staining for Bcl-2 in representative cases of (**A**) normal breast tissue, (**B**) benign breast lesion (fibroadenoma), (**C**) ductal carcinoma, and (**D**) invasive lobular. The scale bar is 50 µm.

We performed an immunohistochemical analysis using SGK1 and Bcl-2 antibodies on tissue microarrays of breast tissue samples. Representative images of all immunohistochemical markers are shown in Figure 2 and Figure 3. The clinical and demographic data for this subset are summarized in Table 2. Our cohort comprised 111 nH Black, 86 nH White, and 83 Hispanic breast cancer cases. Overall, the study population had a mean age, at diagnosis, of 55.9 (SD ± 10.9) years, and the majority were menopausal (83%), overweight, or obese (82%). Valid samples included breast cancers of various subtypes and stages of tumor progression. Most cases were of the ductal type (76%), 58% were diagnosed at a late stage, 61% were low/intermediate grade, and 77% were positive for ER or PR. Immunohistochemical subtyping has shown that most of our cases were Luminal A (68%), and 18% had a triple-negative phenotype.

### 3.2. Increased Expression of SGK1 in Breast Cancer Tissue

We observed diffuse cytoplasmic staining in normal breast tissue and fibroadenomas (Figure 2). SGK1 lacks the exclusive myoepithelial staining pattern we previously reported for GCR [13]. Cytoplasmic staining was detected in all histological and molecular subtypes of breast cancer tissue. However, the mean H score and the percentage positively stained were lower in normal breast tissues compared to the tumor and fibroadenoma samples; this difference was statistically significant. We observed the same upregulation of cytoplasmic SGK1 in fibroadenoma and all subtypes of breast cancer tissue, compared to normal tissue, when SGK1 was categorized according to the median H score (low < 30 and high 30) (Table 2). With this categorization, only 25% of normal tissues were strongly positive for SGK1 compared to 50% among fibroadenoma and 53% among breast cancer tissue. SGK1 expression varied between histological and molecular breast cancer subtypes. The highest mean H score was among subtypes associated with a poor prognosis and low survival, such as hormone receptor negative (mean H score = 58), triple-negative (mean H score = 56), Her2 + (mean H score = 56), and mixed/other types (mean H score = 52).

High expression of SGK1 was associated with the ER-/PR- status (*p* = 0.031). High SGK1 staining was associated with lower odds of ER+ and/or PR+ status (OR 0.54, 95% CI 0.29–0.97). Adjusting for potential confounders, such as age, self-reported race/ethnicity, stage, and grade at diagnosis did not change the point estimate, but the confidence interval increased and included one (OR 0.6, 95% CI 0.3–1.3).

### 3.3. Decreased Expression of Bcl-2 Expression in Breast Cancer

Figure 3 presents a representative case showing Bcl-2 staining in breast tissue. Bcl-2 staining was invariably cytosolic. We observed intense cytoplasmic staining of the myoepithelial and luminal layers in normal breast tissue and fibroadenomas.

**Figure 4:**
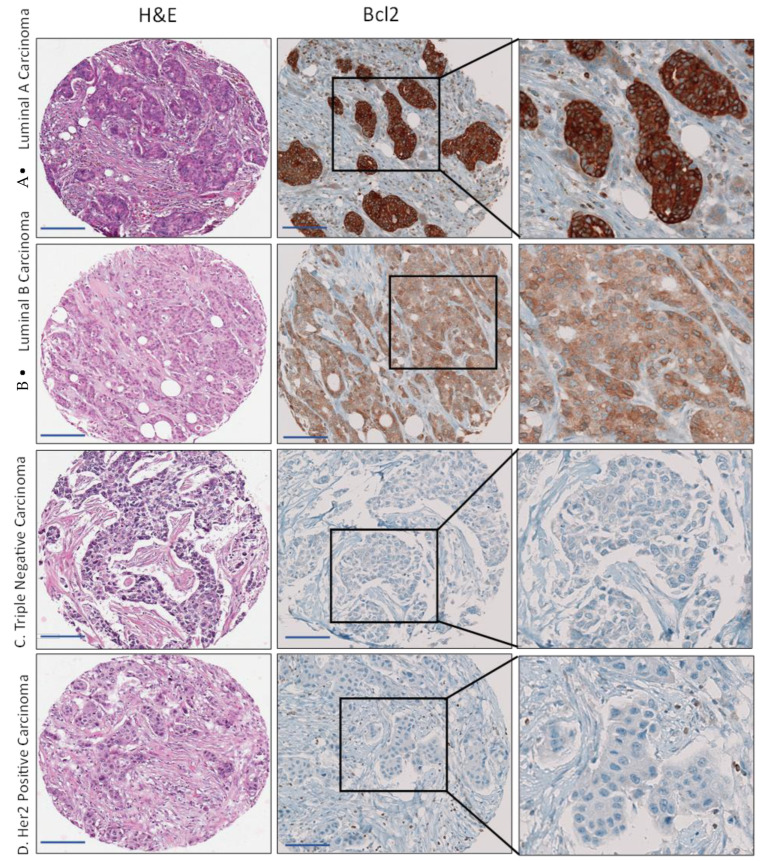
Immunohistochemical staining for Bcl-2 in the representative cases of (**A**) Luminal A, (**B**) Luminal B, (**C**) triple-negative, and (**D**) Her2 positive carcinoma. The scale bar is 50 µm.

We also detected cytoplasmic staining in breast cancer tissue in all histological and molecular subtypes. There was a statistically significant decrease in the mean score and the percentage positively stained between normal and fibroadenoma samples compared to tumor breast tissue (Table 3). Bcl-2 expression varied between histological or molecular subtypes of breast cancer. The lowest mean H score was among subtypes associated with a poor prognosis and low survival, such as the estrogen receptor-negative (0.3), triple-negative (0.4), and Her2+ (0.1) breast cancer subtypes. High expression of Bcl-2 was associated with ER+/PR+ status (*p* = 0.031), Her2+ (*p* < 0.0001), and high GCR (*p* = 0.024). Figure 4 illustrates the decrease in Bcl-2 expression in aggressive breast cancer subtypes, such as triple negative cases and Her2 negative cases, compared to Luminal A. We observed the same pattern of cytoplasmic Bcl-2 expression among our samples when we classified the Bcl-2 score according to the median score (low = 0 and high > 0). We detected Bcl-2 staining in 79% of breast cancer tissues compared to 100% for fibroadenoma and normal tissue.

### 3.4. SGK1, Bcl-2 Expression, and Clinicopathological Characteristics of Breast Cancer

Next, we examined the baseline characteristics of the study population according to SGK1 and Bcl-2 staining (Table 4). Although there was no statistical difference in mean Bcl-2 expression by race/ethnicity, the proportion of tumors with high expression of Bcl-2 was associated with self-reported race/ethnicity (*p* = 0.005). Specifically, mean Bcl-2 expression was greater for nH Black patients than nH White patients (70% vs. 89%, *p* = 0.005). Bcl-2 expression was greater for patients with greater BMI, and diagnosed greater Bcl-2 expression was associated with an ER/PR positive disease, low histological grade, early stage, and Her2+ disease. Bcl-2 expression was not associated with age at diagnosis, a family history of breast cancer, or menopausal status. With respect to SGK1 expression, there were no differences in SGK1 staining associated with self-reported races/ethnicity, age at diagnosis, stage and grade at diagnosis, BMI, or menopausal status.

**Figure 5:**
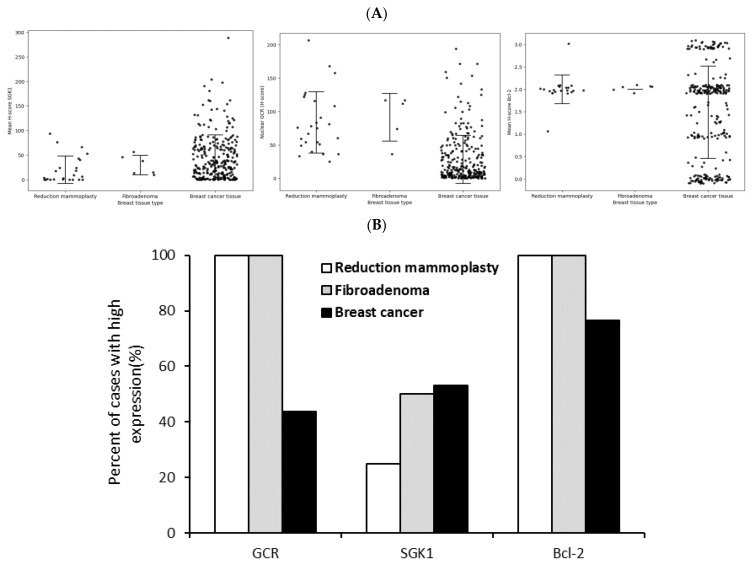
Lower GCR, higher SGK1, and Bcl-2 staining in cancer tissue compared to non-cancer breast tissue. The mean H scores (**A**) and percentages (**B**) of cases with high expression of SGK1 and Bcl-2 in breast cancer vs. noncancerous tissue (Reduction mammoplasty and fibroadenoma).

The main objective of this study is to evaluate the expression of SGK1 and Bcl-2 in breast cancer tissue. Among the BCCC subcohort, the expression of SGK1 was reduced, while that of Bcl-2 increased (Figure 5). We observed that the staining of fibroadenoma was similar to cancer tissue in SGK1 staining, but it was also similar to normal tissue in Bcl-2 staining.

### 3.5. Expression of SGK1 and Bcl-2 and Breast Cancer Survival

**Figure 6:**
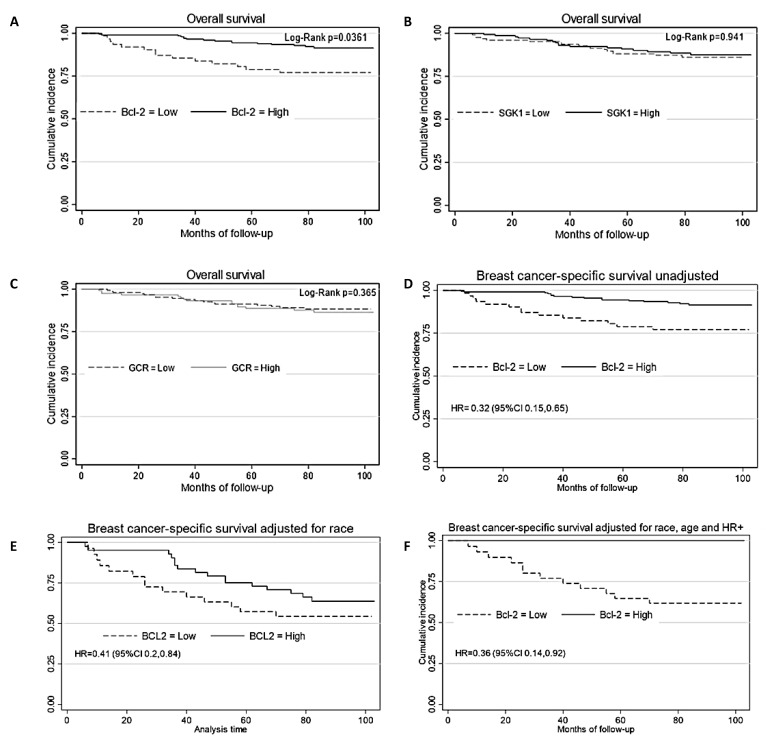
The Kaplan–Meier curve of cumulative overall survival, according to Bcl-2 (**A**), SGK1 (**B**), and GCR (**C**) staining. The Kaplan–Meier curve of breast cancer-specific survival, according to Bcl-2 staining unadjusted (**D**), is adjusted for self-reported race/ethnicity (**E**), adjusted for race/ethnicity, age, and Hormone receptor positive (**F**).

We also evaluated the correlation of GCR, SGK1, and Bcl-2 with overall survival and breast cancer-specific survival. Data from 263 cases for Bcl-2, 266 cases for GCR, and 271 cases for SGK1 were available for this analysis. The median follow-up time was 79 months (a range of 6 to 103 months). During the follow-up period, 52 cases of death from any cause and 43 deaths from breast cancer were recorded. Cases with high Bcl-2 have a higher overall survival rate compared to cases with low Bcl-2 (log-rank *p* = 0.0478) (Figure 6A) and breast cancer-specific survival (log-rank *p* = 0.0025) (Figure 6D). In the Kaplan–Meier analysis, patients with low Bcl-2 expression had a significantly lower survival probability than those with high Bcl-2 expression (HR 0.53, 95% CI 0.29, 0.97). Increased expression of Bcl-2 was associated with a protective effect on breast cancer-specific survival (HR 0.32, 95% CI 0.16, 0.65). High expression of Bcl-2 remained strongly associated with breast cancer survival after an adjustment for race (HR 0.41, 95% CI 0.2, 0.85). The expression of BCL-2 remained strongly positively associated with protection against breast cancer death, with an additional adjustment for age and ER+ or PR+ status (HR = 0.36, 95% CI 0.14, 0.92). SGK1 and GCR staining was not related to overall or breast cancer-specific survival.

## 4. Discussion

Due to the inherent heterogeneity of breast cancer, scientists have yet to identify specific markers that help distinguish breast cancer subtypes and predict prognosis and treatment options. The Breast Cancer Care in Chicago (BCCC) aims to investigate the biological bases for the racial/ethnic disparity in breast cancer incidence and outcome.

Several epidemiological studies have shown that the cellular alterations resulting from chronic psychosocial stress may increase breast cancer development and progression. Among the primary mediators of stress is glucocorticoid, which acts via its cytoplasmic receptor, the glucocorticoid receptor (GCR). Glucocorticoids, signaling via the GCR, regulate several physiological and pathological processes in breast tissue through interactions with other proteins, such as SGK1 and Bcl-2. Analyzing the current BCCC cohort, we have previously shown that GCR is downregulated in breast tissue compared to normal tissue. Here, we examined SGK1 and Bcl-2 protein expression in respective breast cancer tissue microarrays. The originality of this paper originates from our attempt to establish the predictive status of GCR, SGK1, and Bcl-2 in the BCCC cohort, which includes a multiethnic population of incident breast cancer cases with linked clinical, genetic ancestry, survival, and sociodemographic data, as well as histologic and molecular subtyping.

Tumor development is a multistep process that includes dysregulated energy metabolism, sustained proliferation, apoptosis evasion, and metastasis; GCR, SGK1, and Bcl-2 have been associated with these processes. However, no research has been done to determine the correlation between the expression patterns of these proteins in breast cancer tissues. We have previously shown that GCR is reduced in breast cancer tissue compared to non-cancerous breast tissues [13]. Here, we used the same series of breast cancer cases, from a multi-racial population with defined clinical characteristics and survival data, to measure the protein expression of SGK1 and Bcl-2.

Significant findings in this study are: (1) Compared to normal tumor tissues, SGK1 protein expression was higher in breast cancer tissues, especially in ER/PR negative and triple-negative tumors; (2) Bcl-2 protein expression was lower in breast cancer than normal breast tissues; (3) higher Bcl-2 expression was associated with hormone receptor positivity, lower tumor grade, and earlier stages; longer survival (4) Bcl-2 protein expression was lower in women who self-reported as African American compared to Hispanic and nH White women.

SGK1, a serine/threonine that is kinase-dependent on phosphatidylinositol 3-kinase, is expressed in many tissue types and induced by several hormones, such as glucocorticoids and androgens [22]. SGK1 has been shown to regulate glucose levels [23], affect various physiological functions, and plays an active role in the pathophysiology of obesity, diabetes, autoimmune diseases, and cancer [23]. SGK1 expression is upregulated in some tumors, such as breast cancer [24], multiple myelomas [25], and lung cancer, and it is downregulated in others, such as prostate cancer [26]. SGK1 acts as an anti-apoptotic factor promoting cell survival signal and cell cycle progression [27]. SGK1 has also been shown to contribute to tumor development and progression and affect response to treatment [28].

SGK1 was expressed, mainly, in the cytoplasmic compartment, which is consistent with the pattern of expression previously reported [24,29]. Our results showed an increase in the expression of SGK1 in breast cancer tissue compared to benign tissue. This strong cytoplasmic expression of SGK1 was associated with a negative ER/PR status, but it was not associated with race/ethnicity, age at diagnosis, stage or grade at diagnosis, or molecular subtypes of breast cancer. As expected, GCR expression was positively correlated with SGK1 expression in breast cancer tissue, as glucocorticoids induce the SGK1 protein expression. SGK1 expression in breast cancer has previously been examined in a small number of breast cancer cases. Sahoo et al. [24] found that 19 of 40 tumors from 37 patients had positive SGK1 staining, with the majority showing exclusive cytoplasmic subcellular localization. Zhang et al. [29] used a multi-tumor tissue microarray from the Tissue Array Research Program (TARP-2) to find low or undetectable SGK1 in normal breast tissues (5/5) and high SGK1 in most breast cancer tissues (29/38). Tumor cells up-regulate and down-regulate gene expression to help them grow and metastasize. We observed an up-regulation of the expression of the SGK1 protein, regardless of the histological type, stage, or grade of breast cancer, indicating an oncogenic role of SGK1 early in tumorigenesis in breast cancer.

The molecular mechanisms underlying the association between higher SGK1 expression and breast cancer tissues is not clear. Animal studies have shown that chronic caloric restriction was correlated with increased glucocorticoid-induced SGK1, downstream signaling pathways, decreased p53 function, and promoted colonic tumorigenesis [30]. Furthermore, SGK1 knockout mice developed fewer colonic tumors than wild-type mice [31], and inhibiting SGK1 decreased the number of colonic tumors [32,33]. Moreover, activating or upregulating SGK1 was shown to promote breast tumors by downregulating p53 expression or inducing apoptosis similarly to what was reported in colon and prostate cancers.

Apoptosis is an important mechanism in the pathogenesis of breast cancer, and SGK1 was shown to regulate several biological processes in the cell, including apoptosis. SGK1 has been shown to regulate Bcl-2 expression via the transcription factor, Forkhead box protein O1 (FOXO1) [34]. Therefore, we sought to assess the expression levels of Bcl-2 in the current cohort. Bcl-2 belongs to a family of apoptosis-related proteins [35]. Bcl-2 has been shown to promote cell viability without promoting cell proliferation [36]. Interestingly, high Bcl-2 protein expression has been associated with an early grade, slow-proliferating ER+ profile and favorable outcomes in breast cancer, independent of many pathophysiological characteristics such as lymph node status, tumor size, and grade [37]. An intact ER signaling pathway is vital for normal breast cellular function. The paradoxical correlation of Bcl-2 with a good prognosis could be a marker for an intact ER signaling pathway [38].

We observed a decrease in Bcl-2 expression in breast cancer compared to normal breast tissue. Similar to previous reports [39], the downregulation of Bcl-2, among our cases, was more evident in aggressive subtypes of breast cancer, such as ER- and/or PR- (27%), triple-negative (31%), and Her2+ (14%), compared to ER positive BC tissue. The mechanisms by which Bcl-2 can protect against breast cancer, including its role in apoptosis or whether non-apoptotic functions are involved, are yet to be elucidated and correlated.

We observed a correlation between Bcl-2 and GCR expression measured in respective breast tissue microarrays. We previously showed that GCR expression was lower in breast cancer tissue compared with normal breast tissue, regardless of tumor characteristics [13]. The biological interaction between GCR and Bcl-2 is not well understood. However, it has been suggested that GCR modulates Bcl-2 activities directly, or through other modulators, to regulate apoptosis [10,15]. We observed lower expression of both GCR and Bcl-2 in normal breast tissue compared to tissue that had undergone tumorigenesis. Further down-regulation of Bcl-2 in tumor tissue is associated with more aggressive characteristics of breast cancer, such as later stages and higher grades at diagnosis. Reducing the expression of Bcl-2 and GCR might be one of the ways cancer cells create a favorable environment for growth.

Bcl-2 expression was lower for patients who self-reported their race/ethnicity as nH Black compared to nH White or Hispanic. Matias et al. found that the Bcl-2 gene and protein expression were lower in patients with African ancestry than in White patients in triple-negative breast cancer cases [40]. It is also possible that Bcl-2 is an informative marker of ancestry without any role in the pathogenesis of breast cancer, but this seems unlikely given what is known about the function of this protein.

We found that Bcl-2 expression was associated with a higher overall and breast cancer-specific survival, whereas SGK1 and GCR expression did not appear to be associated with survival. Our results are consistent with previous findings, which found that Bcl-2 expression was associated with a better prognosis for breast cancer despite its anti-apoptotic characteristic [20,37,41,42,43]. It is worth noting that a large body of research demonstrates that Bcl-2 protein expression has a greater prognostic value in hormone receptor positive breast cancer than in hormone receptor negative breast cancer [44,45]. Some studies, however, have shown that Bcl-2 expression is an independent prognostic factor, even in hormone receptor-negative or triple-negative breast cancers [46]. As a result, bigger sample size investigations are needed to assess the predictive relevance of Bcl-2 in different hormone receptor expression states.

This investigation provides crucial data regarding the expression levels of GCR, SGK1, and Bcl-2 in respective breast cancer TMAs. Nonetheless, future research is necessary to investigate the underlying molecular mechanisms that these proteins may regulate. The mitochondrial function is at the top of the proposed pathways from previous investigations. Mitochondria play an important role in cancer pathogenesis in general [47]. The molecular mechanisms of mitochondrial dysfunction in breast cancer are not entirely understood, and they might involve crosstalk between GCR, SGK1, and Bcl-2 via chronic stress and apoptosis pathways [48,49,50,51]. Future mechanistic research is required to investigate the functional implications of the differential expression of GCR, SGK1, and Bcl-2 on various molecular mechanisms, including mitochondrial functions.

## 5. Conclusions

Our study of 280 patients with well-characterized breast cancer tissue is the largest study to evaluate the expression of the SGK1 protein in breast cancer as far as we are aware. Our study suggested that Bcl-2 might be differentially (over) expressed for nH Black patients compared with nH White and Hispanic patients. Strengths of this study include the availability of detailed demographic and clinical data on a diverse sample of patients taken from a population-based study that should be generalizable to an urban population of US breast cancer patients. Limitations to this study include its cross-sectional nature, which obscures the direction of associations between protein expression and tumor characteristics. There are also limitations to the tissue microarray and immunohistochemical staining technique. The single-color immunohistochemical technique visualized only a single marker in respective TMAs, and statistical correlation was performed to examine the relationship among our markers. Future studies that examine the co-expression of these markers in the same tissue sample are required to confirm our correlation data. In conclusion, we observed increased cytoplasmic SGK1 and decreased Bcl-2 expression in breast tissue associated with the ER/PR negative status. High Bcl-2 is associated with better breast cancer outcomes. However, the possibility that those high Bcl-2 tumors were less prevalent in nH Black patients’ cases might provide insight into our understanding of the racial/ethnic disparity in breast cancer incidence and outcomes.

## Figures and Tables

**Table 1:** A list of antibodies for immunohistochemical staining.

Antigen	Manufacturer	Host	Clone Number	Dilution	Retrieval Method
GCR	Lecia/Novocastra	Mouse	4H2	1:25	HIER
SGK1	Novus/Biologicals	Rabbit	NB100-92054	1:50	CC1 Mild
Bcl-2	Cell Marque	Mouse	124	Predilute	CC1 Mild
ER	Ventana	Rabbit	SP1	Predilute	CC1 Mild
PR	Ventana	Rabbit	1E2	Predilute	CC1 Mild
Her-2	Ventana	Mouse	4B5	Predilute	CC1 Mild
CK 5/6	DAKO	Mouse	D5 and 16B4	1:50	HIER
EGFR	Ventana	Mouse	3C6	Predilute	CC1 Mild

HIER: heat-induced epitope retrieval; CC1: cell conditioning solution 1.

**Table 2:** The distribution of demographic and tumor-related factors of cases of the BCCC subcohort cases for the TMA study.

	% Cases
**Self-reported race/ethnicity (*n* = 280)**	
nH Blacks	40
nH Whites	30
Hispanics	30
**Age at diagnosis (*n* = 280)**	
Less than 50 years	31
Equal or greater than 50 years	69
**CDC BMI categories of BMI (*n* = 278)**	
Normal weight (18.5–24.9 kg/m^2^)	21
Overweight (≥25 kg/m^2^)	79
**Menopausal (*n* = 279)**	
No	17
Yes	83
**Histological subtypes (*n* = 258)**	
Ductal carcinoma	76
Lobular carcinoma	11
Mixed ductal/lobular carcinoma and Other	13
**Grade (*n* = 272)**	
Low/intermediate	61
High	39
**Stage (*n* = 277)**	
0,1 (early stage)	42
2,3,4 (late stage)	58
**Hormone receptor status (*n* = 276)**	
ER- and PR-	23
ER+ and/or PR+	77

**Table 3:** The distribution of SGK1 and Bcl-2 staining by breast tissue subtypes.

Breast Tissue	N	SGK1 H Score ^a^ Mean, *p*-Value ^d^	High SGK1 ^b^ %, *p*-Value ^e^	N	Bcl-2 H Score ^a^ Mean, *p*-Value ^d^	High Bcl-2 ^c^ %, *p*-Value ^e^
**Reduction mammoplasty**	**24**	**20**	**25**	**21**	**2.0**	100
Fibroadenoma	6	30	50	6	2.0	100
Breast cancer tissue	272	46	53	264	1.5	77
		*p* < 0.0001	*p* = 0.029		*p* = 0.038	*p* = 0.018
**Histological subtypes**						
Ductal carcinoma	195	47	54	195	1.4	71
Lobular carcinoma	28	29	39	28	1.7	93
Mixed & Other	35	52	57	35	1.8	85
		*p* = 0.1044	*p* = 0.287		*p* = 0.038	*p* = 0.017
**Molecular subtypes**						
Luminal A	179	41	49	178	2.0	96
Luminal B	14	44	57	14	1.2	79
Triple Negative	48	56	60	45	0.4	31
Her2	21	56	67	21	0.1	14
		*p* = 0.123	*p* = 0.25		*p* < 0.0001	*p* < 0.0001
**Hormone receptor status**						
ER- and PR-	62	58	66	59	0.3	27
ER+ and/or PR+	202	43	51	201	0.9	92
		*p* = 0.028	*p* = 0.031		*p* < 0.000	*p* = 0.031
**Glucocorticoid Receptor status**						
Low (<17 H score)	148	44	51	147	1.4	73
High (≥17 H score)	140	43	51	135	1.7	84
		*p* = 0.81	*p* = 0.99		*p* = 0.008	*p* = 0.024

^a^ Mean H-score. ^b^ Percentage positivity for SGK1: a tissue was considered positive for cytoplasmic SGK1 when the sample had an H score ≥ 30. ^c^ Percentage positivity for Bcl-2: a tissue was considered positive for Bcl-2 when the sample had a score > 0. ^d^ F-test *p*-value ^e^ Chi-square *p*-value.

**Table 4:** Baseline characteristics of study subjects according to SGK1 and Bcl-2 staining.

Breast Tissue	N	SGK1 H-Score ^a^ Mean, *p*-Value	High SGK1 ^b^ %, *p*-Value	N	Bcl-2 H-Score ^a^ Mean, *p*-Value	High Bcl-2 ^c^ %, *p*-Value
**Self-reported race/ethnicity**						
nH Blacks	118	45	55	112	1.4	70
nH Whites	90	40	51	87	1.7	89
Hispanics	94	45	46	92	1.5	80
		*p* = 0.63	*p* = 0.52		*p* = 0.116 ^d^	*p* = 0.005 ^e^
**Age at diagnosis**						
Less than 50 years	84	52	56	82	1.4	71
Equal or greater than 50 years	188	43	52	182	1.5	79
		*p* = 0.129	*p* = 0.56		*p* = 0.32	*p* = 0.137
**CDC categories of BMI**						
Normal weight (18.5–24.9 kg/m^2^)	56	43	52	56	1.3	66
Overweight/obese (≥25.0 kg/m^2^)	214	47	54	206	1.5	79
		*p* = 0.59	*p* = 0.79		*p* = 0.147	*p* = 0.042
**Menopausal**						
No	46	45	48	46	1.4	70
Yes	225	46	55	217	1.5	78
		*p* = 0.828	*p* = 0.39		*p* = 0.373	*p* = 0.227
**Stage at diagnosis**						
0,1 (early stage)	111	43	53	106	2	90
2,3,4 (late stage)	158	47	53	155	1	68
		*p* = 0.999	*p* = 0.483		*p* < 0.0001	*p* < 0.0001
**Histologic grade**						
Low/intermediate	161	43	52	99	2	90
High	103	51	56	258	1	54
		*p* = 0.483	*p* = 0.511		*p* < 0.0001	*p* < 0.0001

^a^ Mean H-score. ^b^ Percentage positivity for SGK1: a tissue was considered positive for cytoplasmic SGK1 when the sample had an H score ≥ 30. ^c^ Percentage positivity for Bcl-2: a tissue was considered positive for Bcl-2 when the sample had a score > 0. ^d^ F-test *p*-value. ^e^ Chi-square *p*-value.

## Data Availability

The data presented in this study are available on request from the first author (U.A.).
